# Transitions to Long-Term Care for People Living with Dementia: Social Death and Social (Dis) Connections

**DOI:** 10.3390/nursrep15090319

**Published:** 2025-09-03

**Authors:** Veronika Williams, Mary Pat Sullivan, Christina Victor

**Affiliations:** 1 Faculty of Education and Professional Studies, Nipissing University, North Bay, ON P1B 8L7, Canada; marypat.sullivan@nipissingu.ca; 2 College of Health, Medicine and Life Sciences, Department of Health Sciences, Brunel University London, Uxbridge UB8 3PH, UK; christina.victor@brunel.ac.uk

**Keywords:** dementia, long-term care, social death, care transitions, social isolation

## Abstract

**Background/objectives:** Dementia is the most common cause for long-term care placement for people over the age of 65 years. The decision and the transition are often very difficult for families and the type and timing of effective support not well understood. In this exploratory study, we aimed to gain a better understanding of the experience of this transition to a long-term care setting and its impact on social connections. **Methods:** We conducted virtual in-depth interviews with a sample of spouses (N = 5) who had identified their partner’s move to a nursing home as particularly distressing. Interview data were analyzed using thematic analysis. **Results:** We identified that the transition process involved a particular disruption for dyads’ social health, although there was rarely a consideration in planning or support provision. For the person living with dementia, the unacknowledged loss of their social world reinforced their social death. Their grieving partner was explicitly and implicitly encouraged to recover a new social world as a means of healing from the loss. **Conclusions:** Our findings reinforce the need for evidence-informed support during the transition to long-term care for someone living with dementia and their partner.

## 1. Introduction

Dementia is one of the leading causes of morbidity and mortality, and projections indicate that its global prevalence will continue to rise significantly due to ageing populations, with an estimated 139 million people affected by 2050 [1]. Most research on experiences of individuals and families affected by dementia focuses on negative consequences of dementia, such as caregiver burden [2,3,4] and negative psychological and social impacts for both those living with a dementia diagnosis and families [5,6,7].

Whilst there is an increasing body of literature presenting more positive narratives of families affected by dementia, stigma continues to pervade dementia narratives overall and suggests that this contributes significantly to the social isolation experienced by individuals [8,9]. It has also been recognized that social isolation does not only affect the person living with a dementia diagnosis but also their family [10], and the impact of social isolation on the family unit has received particular attention during the COVID-19 pandemic [11,12,13]. Yet the societal stigma surrounding dementia prevails and continues to impact families’ social connections. Nguyen and Li [14] differentiate between public stigma and self-stigma in the context of dementia and found that whilst public stigma was linked to social isolation, self-stigma contributed to individuals and families delaying seeking support. The cognitive decline as a result of the dementia diagnosis and the lack of support, which can be compounded by the delay in seeking support, often leads to a family feeling unable to provide support for the person at home. It is estimated that one third of people diagnosed with dementia and younger than 80 years of age live in a long-term care setting, and nearly 70% of residents in long-term care settings in Canada have a diagnosis of dementia. Long-term care in Ontario is similarly organized as in many other high-income countries, with more than 50% of homes run by for-profit organizations and the remainder provided by non-profits and municipalities [15].

Transitions to long-term care settings are often experienced as stressful, as a move to long-term care for a person with dementia tend to take place at a time of crisis [16,17]. Research has detailed the distress in families feeling upset about the poor level of care their family received and lack of communication with staff during the transition to a long-term care facility [18]. A recent scoping review on interventions to improve transitions to long-term care settings from home for older people (most of whom were diagnosed with dementia) found that most interventions either focus on the older person, the family, or staff and target either the transitional or post-transition phase [19]. Yet it has been argued that interventions to support the transition to a long-term care setting need to not only take account of the complexity of the inseparable care triad (i.e., the person living with dementia, their family, and health care professionals) but also delineate support during the pre-transition, transition, and post-transition phases [17,18,19,20]. Furthermore, a systematic review assessing the effectiveness of interventions to support people living with dementia and their care partner found that all of the included trials focused on interventions supporting the care partner, and as a result of poor methodological quality, the evidence was inconclusive in relation to their effectiveness [21].

While we have considerable evidence on the general experience of a long-term care transition on families, including loss of family, home and social connections, feelings of guilt [22,23], and depression experienced by the family [24,25], there is less focus specifically on the impact on social connection leading up to a move to a long-term care setting on the dyad. In the study reported here, we explored the experience of the transition to a long-term care setting for a person living with dementia from their partner’s perspective and its effect on the dyad’s social connections leading up to, during, and after the transition.

## 2. Materials and Methods

### 2.1. Study Setting

This study was part of a larger Canada-based research project which aimed to investigate the experiences of social isolation and loneliness for people living with dementia and their principal resident care partners in northeastern Ontario. Our results, based on interviews with 19 participants affected by a dementia diagnosis, offered unique insights into interdependent relationships and the experience of social isolation and/or loneliness within dementia care. During the initial analysis, we identified considerable differences in experiences of social connections for people whose partner had been moved to a long-term care setting and thus wanted to explore these further in the sub-study with participants whose partner had moved to a LTC setting presented here.

### 2.2. Ethical Considerations

This study received ethical approval from the Research Ethics Committee, Nipissing University, REF 102650. All participants received additional information on this exploratory study nested within the main research project. We confirmed consent with participants prior to interview (written and/or audio recorded). Participants were provided with contact information for support if required.

### 2.3. Study Design and Participants

We conducted an exploratory qualitative study using semi-structured interviews with the aim to provide a more nuanced understanding of individuals’ experiences of transition to a long-term care setting, and its impact on social connection, in the context of a dementia diagnosis. We purposefully recruited those participants who took part in the main study and whose partner had been moved to a long-term care facility in the previous two years to this exploratory interview study. All participants who were invited agreed to take part in this study on experiences of social connections in the context of a transition to a long-term care facility.

### 2.4. Data Collection and Analysis

A semi-structured interview guide was developed by the research team and focused on the circumstances leading up to the transition, their experience of the transition itself, and the impact on the couple’s social connections throughout. Virtual interviews were conducted by the same research assistant using the Zoom© platform and lasted on average one hour. Fieldnotes were taken during the interview to support the analytical process. Virtual interviews can present some challenges in terms of limiting ability to observe body language and subtle social cues [26]; however, we did not encounter any significant issues. Since our participants were geographically dispersed across Ontario, and we were still experiencing limitations to travel as a result of the COVID-19 pandemic, virtual interviews afforded more flexibility and allowed us to include participants who may have otherwise not been able to take part in this research.

Interview data were transcribed verbatim using the built-in Zoom© 2022 online transcription function, and cleaned transcripts were organized in NVIVO. We used a thematic analysis approach [27], following the six phases of data familiarization, generating initial codes, generating initial themes, reviewing and defining themes, and producing an initial report on results with interpretation. Data analysis was conducted by the first author (VW) and the development and generation of themes were discussed with the co-authors during an iterative and reflexive analysis process. As the analysis progressed, the research team drew on existing theory that was relevant to further interrogate the data and explain participants’ stories.

To ensure trustworthiness [28,29] of findings, we reviewed identified themes with a research member with lived experience and interrogated data iteratively in view of identified initial themes throughout the analysis process: whether we had enough contextual information to interpret the data and whether our interpretations made sense as we progressed with analysis. We kept a research diary of not only field notes but also analytical thoughts and decisions throughout the data collection and analysis processes and included a reflexive account on the analytical process and the researchers’ impact on data and interpretations.

### 2.5. Reflexivity

The research team consisted of an experienced interdisciplinary research team with expertise in dementia, ageing, and loneliness. The data collection was supported by a research assistant with a background in psychology. The first author kept a reflexive journal during the analytic process and regularly discussed the identified themes with the second author, not only to challenge assumptions but also to consider alternative perspectives and theoretical links.

## 3. Results

We identified several themes that related to the experience of social connections and the transition to a long-term care setting: the disruption of social health, maintaining and regaining social connections during this period; benevolent deception in the context of disclosure of the move to the person diagnosed with dementia; and reinforcement of social death, whereby people living with dementia are viewed and treated as if they are no longer part of society and our social lives even though they are still physically alive.

### 3.1. Participant Details

Nineteen participants took part in the main study, and of these, five participants met the additional eligibility criteria (with a partner living in a LTC setting) and agreed to take part in this exploratory study on long-term care transitions. As shown in Table 1, participants’ ages ranged from 58 to 79 years old; three of the participants were female and two were male. Their partners had been transferred to a long-term care setting between two years and four months prior; they were diagnosed with one of the following: young-onset Alzheimer’s Disease, Alzheimer’s Disease, and vascular dementia; length of diagnosis was between 3 and 10 years, although all reported that symptoms had started a considerable time prior to the formal diagnosis. Participants were all located in Ontario, Canada.

We identified three main themes that related to the experience of social connections and the transition to a long-term care setting: the disruption of social health, maintaining and regaining social connections during this period; benevolent deception in the context of disclosure of the move to the person diagnosed with dementia; and reinforcement of social death, whereby people living with dementia are viewed and treated as if they are no longer part of society and our social lives even though they are still physically alive.

### 3.2. Disruption of Social Health, Maintaining and Regaining Social Connections

Participants described the disruption of their social connections as a result of the dementia diagnosis, affecting the dyad and how they tried to maintain old and gain new social connections as a result of both the dementia diagnosis and move to the LTC setting. Whilst for most participants the diagnosis and transition to the LTC setting presented a significant disruption to social health and connections, they would also explain how they would try and maintain social connections with others and between each other as a couple by making adjustments. For some, the move of the spouse to a LTC setting also presented an opportunity for new social connections without their partner.

Andrew, whose wife Maggie had been diagnosed with young onset Alzheimer’s Disease 10 years prior, explained how she preferred quieter environments and interactions as a result of the diagnosis. This was particularly apparent as symptoms progressed and he explained how this affected their ability to spend time with friends as a couple: “*I couldn’t take her up to a big group, or a party of 10 people at someone’s home, …. she could never manage that. We started to probably spend more and more time by ourselves … and other people that were friends with us kind of went on with their lives.*”

However, people’s discomfort with the symptoms of dementia contributed to their withdrawal from social interactions with Andrew and Maggie: “*the social disconnection, I should say, started years before she went into … long-term care … her behavior, because of her understanding, her ability to communicate with people. And people were uncomfortable, you know, with it*”. This social disconnection continued once Maggie had moved to a long-term care facility and only close relatives would visit Maggie in the home, and since Andrew spent most days of the week with her, continued to experienced disruptions to his social connections: “*I still feel that I don’t have those connections.*” Maggie was unable to communicate verbally and lost the cognitive ability to participate in any of the activities offered at the nursing home. Andrew reflected on maintaining their connection: “*She still can walk. And she does that generally, if I get her up. And then I hold her hand, and then we’ll walk. And that’s kind of our social connection*”.

Lynette’s husband Peter had moved to a long-term care home a few months prior to the interview, and although she mentioned having difficulty in engaging in the same activities as a couple prior to the move, the main social disruption occurred as a result of the transition and how this was handled by various organizations, who recommended a total social disconnect for some time immediately after the move. “*I was told not to go in for two weeks to see him, when he first went in, to give him time…The [organization] That’s what they suggested. But, I… I, I couldn’t do that. I had to go. I went every day. And, and a lot of that, had to do, of course, because of, isolation with Alzheimer’s. So, I, I could not let him go and just be there. But… it would have killed him. So, it was, again, a very stressful time.*” This experience was shared by Stephen after his wife Emily was moved to a nursing home: “*When I was taking some courses through the [organization], they mention that when you drop them off … try not to visit right away and too often, so that they get used to the place where they are. Don’t take them home right away, if you’re taking them out. We did take her out for the birthday, but not too often so that she doesn’t expect to be leaving the home… I’d say, I cried for two days straight, I was heartbroken, I had to leave her there and see her the way she was*”.

Once partners had moved to the long-term care setting, social connections for the dyad seemed to diverge for some couples. For the partner in the long-term care home, social connections appear to remain disrupted and lacking, whereas for some of the partner at home, their social connections were revived. Rosemary described her husband Michael’s experience of his social connections in the nursing home: “*… he doesn’t really have any social connection with anyone there…. I don’t think he has any social connections, even with the visitors. He’s … pretty much on his own…*” compared to her own connections that were growing: “*I definitely started doing more things without him. Joined a few groups. I’ve taken some lessons and courses just for myself. I’ve gone back to work. I’ve traveled with my mom. I spend weekends with friends now, which I wouldn’t have done in the earlier days. I’m carving out a little more of a life for myself without him*”. Similarly, Lynette talked about her ability to reconnect with others once her husband had been moved to a long-term care setting: “*Well, for one thing, I’m never bored. I get up in the morning. I have some stretches and exercises I do. I have family here. I have three siblings here. I talk to them. Speak with my friends. I get in touch with my daughters every day. The day is full. Last night I didn’t get to bed until 11 o’clock. I had a friend for supper. Friends dropped in after she left. My days are full.*” 

Whilst a long-term care setting might have provided an opportunity to make new social connections, participants’ stories did not indicate this. However, their ability to connect with others was likely largely impacted by the COVID-19 pandemic, which made any contact or even visiting as a family impossible.

### 3.3. Benevolent Deception During Transition

Participants described the process of transition to the long-term care setting for their partner as stressful and largely unsupported, particularly in relation to explaining the situation to their partner. These stories shared a sense of benevolent deception whereby the partner with dementia was not told where they were going and why, with the assumption that this would upset them and as a result they might behave unpredictably.

Stephen explained how his wife might have experienced the transition: “*I think the first, the first little while was tough on her, I believe, because she didn’t know exactly why she was there. And then, she was saying, I’m hoping I’m going to get better and come home. And she knew that something was wrong, but didn’t know exactly what.*” Similarly, Lynette told a similar story when they prepared for Peter to move to a nursing home: “*We had to move [his things] from the house for him, trying to do this without telling him. We couldn’t tell him right away, because I didn’t know how he’d react, going to the nursing home.*”

Christine, whose husband Daniel had been in living in a nursing home for almost a year at time of the interview recalled the day of moving him to the home and how the family withheld information from Daniel on what was happening: *“of course we took him in* [nursing home] *under false circumstances for him. He thought he was going to see our family doctor and it was just a way to get him in there because he was very much not accepting any of this. He didn’t think there was anything wrong with him, so it was done very secretly with my daughter and I. When we got him in there, he was okay ‘til he found this… all these people standing in a circle. And then they wanted to go upstairs in the elevator, so we did that, and he kept saying “where are we going” and “why are we here” and of course, we didn’t talk about anything till we got to the conference room. And then when he… We were talking. He finally looked at me, he says, “is this the […] home? Why am I? What do you? Why am I here?”*

None of the participants talked about alternative advice given to prepare their spouses for the transition to the long-term care setting.

### 3.4. Reinforcement of Social Death

Participants shared their experience on not only how their social connections were dis- and interrupted as a result of both the dementia diagnosis and the transition to a long-term care setting but also how this affected their relationship as a couple.

Participants described the impact on their relationship and a sense of no longer being a couple and their partner being ‘alive but gone’, akin to having socially died. Rosemary described her ‘single’ status after Michael moved to a nursing home: “*suddenly, I wasn’t part of a couple, so much or so obviously. And I really, I really noticed that.*” Christine explained her sense of lost couplehood more explicitly: “*Well, it’s kind of like when somebody dies … because you’re not a couple anymore. You’re single*”. Lynette recalled this experience, particularly during the first year of her husband moving to a nursing home. “*He was gone, but he wasn’t gone. He wasn’t with me anymore. He was somewhere else, but he’s still, part of me. He wasn’t dead. He’s very much alive, but I felt he was gone, but, part of me… it’s like a lost limb, lost part of who I was.*”

However, not all participants shared this sense of lost couplehood and social death of their partner. Andrew, whilst acknowledging the impact of the diagnosis and Maggie’s move to the nursing home had on their social connections, reflected on their continued relationship as a couple: “*there certainly was a loss … I go out there four days a week … I’m there by 9:30 in the morning, and I stay till after she [goes to sleep] … I lay her down to go to sleep, I feed her, and I change her, and clean her, and, and walk with her, and try to provide some activation for her four days a week. … because I’m married, our relationship is so very different than it always was our whole life. She’s in a different location… And people can’t relate to that.*”

## 4. Discussion

This exploratory, descriptive qualitative study aimed to provide insights into the experiences of transitions to a long-term care setting in relation to social connections for individuals and their partners affected by dementia.

Unsurprisingly, participants’ stories reflected on the disruption to social connections and social health as a result of the dementia diagnosis, similarly to existing literature [20,30,31]. The transition process to a long-term care setting was experienced as stressful and uncertain, and whilst this is acknowledged in the literature, it is evident that we are lacking evidence of informed support for families [21,30]. Whilst many nursing home and other organizations provide advice, for example when and how often to visit a family member with dementia, there is dearth of reliable and credible evidence to support this approach [21]. However, some research suggests that nursing home residents with a diagnosis of dementia are less likely to receive visits than other nursing home residents [32], yet post-admission frequency of visits and particularly communication were positively associated with preventing severity of behavioral and psychological symptoms of dementia [33].

The COVID-19 pandemic and resulting lockdown measures significantly impacted on social connections for people living in long-term care settings, leading to emotional and psychological distress [34,35], and was particularly detrimental for people living with a cognitive impairment such as dementia [36] and their families [37]. Given that we collected data during 2022, when lockdowns were still implemented in long-term care, this will have had an additional impact on participants’ and their spouses’ ability to connect with families and other residents in the care setting. The pandemic thus underscored the critical importance of maintaining social connections in long-term care setting for families affected by dementia to maintain psychological and physical health [38], yet it could be argued that the urgency of implementing significant changes has since waned again.

Much has been written about the concept of social death in dementia, whereby individuals are treated as if they are no longer part of society despite being physically alive [39], and the negative impact of thus on their social connectedness [40]. When a person moves to a nursing home, this is further reinforced as the individual with dementia no longer participates in everyday social life, and in fact they are often excluded from social interactions and decision-making processes [41]. The loss of couplehood in the context of dementia has been documented elsewhere and relates to a loss of shared past and shared future [42], loss of everyday activities and meaningful communication [43], and loss of intimate relationship [44]. This is further emphasized when the couple is physically separated due to the person with dementia moving to a long-term care setting and a lack of focus on maintaining the partnership during this transition [45] and resulting loneliness [42]. The impact of one spouse living in a long-term care setting has been described as “married widowhood” [46] and it impacts in terms of changed social lives, though in unequal ways, on both partners. Yet this is not inevitable, and increasingly research considers ‘dignity’ in couplehood in the context of dementia and the importance of maintaining the spousal role rather than being defined by the carer’s role [47,48,49]. However, it is evident that there is a lack of evidence-informed support for couplehood during the transition to long-term care, resulting in a sense of loss, grief, and negative impact on identity and connection [45]. This was reflected in our findings, as participants felt no longer part of a couple and torn between their spouse still being alive, yet gone, with their everyday life starting to resemble more that of a single person. Our findings show that social death might also further be reinforced by long-term care and other organizations through advice regarding limiting or delaying visitation. Whilst this information is not provided in publicly available documentation, nor is it underpinned by evidence, it seems that this advice is provided to some families with a focus on visiting less rather than more often (some organizations refer to ‘weaning yourself and your loved one’). Given that dementia is already a highly stigmatized condition [14,50] and family members and friends often disconnect from a person following a dementia diagnosis and as symptoms worsen [51,52], institutional advice to limit visitation further reinforces the social death of the individual.

Benevolent deception involves the use of ‘white lies’ or misleading information with the intention of protecting or comforting someone. In the care of individuals with dementia, benevolent deception is often employed to manage difficult behaviors, reduce anxiety, or avoid distressing situations. While benevolent deception can be seen as a compassionate approach, it raises ethical questions about honesty, autonomy, and respect for the individual’s dignity. While it has been argued that benevolent deception can be a tool to mitigate the impact of dementia symptoms by maintaining a sense of normalcy and reducing distress [53], it can further contribute to social death by perpetuating the notion that individuals with dementia are incapable of handling the truth or participating in meaningful conversations [54]. There is a fine line between the seemingly good intentions of benevolent deception and its misuse, which may further cause distress to the person with dementia and their families alike [53]. Similarly to previous research, we found that benevolent deception further reinforced social death as the person with dementia is seen as no longer capable of understanding what is happening to them and around them and they are now moved to an institution away from society. Some participants shared that they received informal advice provided by some organizations to limit visitation and connection initially or ‘slowly wean them off regular contact’. However, there is no evidence for this approach, and in fact maintaining regular contact with the person in long-term care and connection during and after the transition has been found to be crucial to supporting well-being and reducing behavioral symptoms [20,21,55,56].

There are some limitations to this study that should be noted in the context of the interpretation of our findings. Since our study was exploratory in nature, it was limited in sample size and geography, which may have impacted the findings. Additionally, we conducted interviews during the COVID-19 pandemic, and this impacted on our ability to recruit participants to the main study (and thus to the exploratory study presented here). The pandemic may have also impacted on participants’ ability to gain new and maintain existing social connections, given our extensive knowledge on the isolating consequences of the lockdowns in LTC settings. We interviewed participants some time after the transition to a long-term care setting; thus, narratives represent retrospective views on this time. Whilst there was some dementia diversity in our sample, this was limited, and thus we do not know if experiences and support needs differ for families with other, rare dementia diagnoses, for example, posterior cortical atrophy (PCA), frontotemporal dementia (FTD), or Lewy body dementia (LBD). Participants made reference to limitations increasing their ability to make new connections within the LTC setting; however, their narratives centered on connections with each other and the world outside the LTC setting.

## 5. Conclusions

This study identified that the transition to long-term care contributes to disruptions of social connections between the person living with a dementia diagnosis and their family and friends, as well as their partner. The reinforcement of social death as a result of the dementia diagnosis and worsened by the move to a long-term care facility was identified as a significant factor in these social disconnections, and the transition itself was not discussed with the partner, thus leading to a sense of benevolent deception.

In conclusion, effective support for families affected by dementia through transitions to long-term care should be informed by evidence-informed, family-centered approaches that recognize the complexity and individual needs of the person living with dementia as well as their family members. Central to this support is the need to maintain social connectedness for both the individual and their family or care dyad, as this connection plays a critical role in emotional well-being. Moreover, tailored support and education must be provided not only in preparation for the move but also throughout the transition and after relocation to a long-term care setting. Future research should focus on identifying family-centered and effective tailored support at different stages of the transition to a long-term care setting for families affected by different types of dementia diagnoses.

## Figures and Tables

**Table 1:** Participant socio-demographic background.

Participant Name *	Age	Sex	Spouse’s Name *, Diagnosis	Time Since Diagnosis	Time Since Move to LTC	Ethnic Background
Andrew	58 years	Male	Maggie, young-onset dementia	10 years	2 years	White
Lynette	79 years	Female	Peter, Alzheimer’s Disease	7 years	<6 months	White, French Canadian
Rosemary	Data not available	Female	Michael, young-onset Alzheimer’s Disease	Preferred not to answer	<6 months	White
Christine	71 years	Female	Daniel, vascular dementia	3 years	12 months	White
Stephen	72 years	Male	Emily, young-onset Alzheimer’s Disease	8 years	18 months	First Nations

* Pseudonyms.

## Data Availability

The datasets presented in this article are not readily available due to ethical reasons, given the small, qualitative data set and potential for identifiable information.

## Public Involvement Statement

There was no public involvement in any aspect of this research.

## Guidelines and Standards Statement

This manuscript was drafted against the COREQ (Consolidated Criteria for Reporting Qualitative Research) for qualitative research. A complete list of reporting guidelines can be accessed via the equator network: https://www.equator-network.org/.
